# Feline panleukopenia-associated clinicopathological abnormalities: first evaluation of diagnostic and prognostic roles of endothelial glycocalyx degradation biomarkers

**DOI:** 10.1080/01652176.2025.2573815

**Published:** 2025-10-14

**Authors:** Amir Naseri, Merve Ider, Busra Burcu Erol, Suleyman Serhat Iyıgun, Murat Kaan Durgut, Mahmut Ok, Hatice Betul Sahin, Nuri Kaan Donmez, Ahmet Icigen, Tunahan Yavuz

**Affiliations:** Faculty of Veterinary Medicine, Department of Internal Medicine, Selcuk University, Konya, Turkiye

**Keywords:** Endothelial glycocalyx, panleukopenia, biomarker, mortality, cat

## Abstract

The aim of this study was to investigate the role of endothelial activation and glycocalyx degradation in the pathogenesis of feline panleukopenia (FPL) using biomarkers and to determine the diagnostic and prognostic significance of these biomarkers. Thirty cats with FPL and 10 healthy cats were enrolled. Clinical examination, blood gases, and complete blood count (CBC) were performed at enrollment. Endothelin-1 (ET-1), asymmetric dimethylarginine (ADMA), vascular endothelial growth factor-A (VEGF-A), and syndecan-1 (Syn-1) concentrations were measured using feline specific commercial enzyme-linked immunosorbent assay (ELISA) kits to assess endothelial glycocalyx damage. Nineteen (63.3%) of the cats with FPL recovered and survived, while 11 (36.7%) died. In cats with panleukopenia, acidemia was the most important blood gas finding, while leukopenia, lymphopenia, monocytopenia, granulocytopenia, and thrombocytopenia were the most dominant CBC findings. ET-1, ADMA, VEGF-A, and Syn-1 concentrations were significantly higher in cats with panleukopenia (*p* < 0.01). Serum Syn-1 and ET-1 concentrations were found to be useful in predicting mortality. In conclusion, the fact that the concentrations of all endothelial glycocalyx biomarkers (ET-1, ADMA, VEGF-A, Syn-1) were higher in cats with panleukopenia compared to healthy cats suggests that endothelial glycocalyx damage occurs during panleukopenia infection. In addition, Syn-1 and ET-1 were found to be potential prognostic factors with high sensitivity and specificity.

## Introduction

Feline panleukopenia (FPL) is a highly contagious, fatal, and oldest known viral disease of domestic and wild cats (Porporato et al. 2018; Gulersoy et al. 2023a). FPL is caused by either feline parvovirus (FPV) or canine parvovirus type 2 (CPV-2) antigenic variants. Both viruses belong to the same species, *Protoparvovirus carnivoran 1*, within the genus *Protoparvovirus* and family Parvoviridae (Walker et al. 2022). Although the incidence of the disease has decreased with vaccination, it is still widespread worldwide and causes sporadic outbreaks (Barrs 2019). The severity of FPV infection depends on immunity, co-infections, and the degree of hematologic abnormalities (especially leukopenia), and clinical disease ranges from subclinical infection to a peracute syndrome leading to sudden death (Kruse et al. 2010). FPL is associated with high case-fatality rates (33%–49%), with death typically resulting from complications such as sepsis, dehydration, or disseminated intravascular coagulopathy (DIC). In fatal cases, the mean survival time from diagnosis to death is reported to range from 2 to 3.5 days, underscoring the need for early diagnosis and intensive supportive care (Stuetzer and Hartmann, 2014; Barrs 2019; Petini et al. 2020; Safwat et al. 2024).

Since FPV infection can cause severe leukopenia and sepsis, there is a potential for widespread vascular injury, including damage to the endothelial glycocalyx (eGCx). The eGCx is a complex mesh of membrane-bound proteoglycans, glycoproteins, and plasma-derived components covering the endothelial surface, where it plays a critical role in maintaining vascular integrity and regulating nitric oxide (NO), dependent signaling (Yen et al. 2015; Weinbaum et al. 2021). In systemic inflammatory states, such as those that can occur in severe FPV infection, proteases including matrix metalloproteinases, thrombin, and plasmin degrade the glycocalyx, leading to endothelial dysfunction (Zhang et al. 2018).

Several markers have been used to assess eGCx integrity including Endothelin-1 (ET-1), Asymmetric dimethylarginine (ADMA), Vascular endothelial growth factor (VEGF-A), and Syndecan-1 (Syn-1). ET-1 and ADMA are inhibitors of nitric oxide synthase (NOs), and their elevated levels have been linked to impaired endothelial function and glycocalyx damage (Davis et al. 2011; Ider et al. 2024). VEGF-A is an endothelial growth factor involved in angiogenesis under both physiological and pathological conditions, and altered circulating VEGF-A concentrations may indicate impaired endothelial NO production (Tang et al. 2022; Ider et al. 2024). Syn-1, a key proteoglycan component of the glycocalyx, is shed into the circulation upon glycocalyx degradation and is considered a sensitive indicator of endothelial injury (Sallisalmi et al. 2012; Bush et al. 2021; Ider et al. 2024).

Numerous studies have reported that endothelial activation occurs at the onset of inflammatory conditions, that the eGCx is the target site, and that the disruption of the glycocalyx contributes to the pathogenesis of disease. This study was designed with the hypothesis that glycocalyx damage, which plays a role in the pathogenesis of various diseases, may also develop in panleukopenia infections in cats. Although endothelial damage biomarkers have been investigated in canine parvoviral infections (Naseri et al. 2020), no studies to date have evaluated these markers in cats with FPL, making this the first report of its kind. In this study, we aimed to evaluate the role of endothelial activation and glycocalyx degradation in the pathogenesis of FPL using biomarkers (ET-1, ADMA, VEGF-A, and Syn-1) and to determine the diagnostic and prognostic significance of these biomarkers.

## Materials and methods

The study was conducted between January 2023 and April 2024 at the Department of Internal Medicine, Faculty of Veterinary Medicine, Selcuk University, Konya, Türkiye.

### Study population and blood sampling

The panleukopenia group in this study consisted of 30 cats of various breeds, ages, and sexes that were admitted to the Selcuk University Faculty of Veterinary Medicine Animal Hospital. These cats exhibited clinical signs of FPV infection, such as lethargy, depression, and diarrhea, and tested positive for fecal FPV antigen. Cats diagnosed with any condition other than panleukopenia were excluded from the study.

The control group comprised 10 healthy owned cats of different breeds, ages, and sexes, presented to the same hospital for routine vaccination, antiparasitic treatment, or general health check-ups. These cats were evaluated as healthy based on the absence of clinical signs, negative fecal FPV antigen test results, normal rectal body temperature, no history of antimicrobial or other drug use within the previous two weeks, and unremarkable findings in complete blood count (CBC), blood gas analysis, and biochemical tests.

All cats were screened for fecal FPV antigens using a commercially available rapid test kit (Vcheck FPV Ag, BIONOTE, Korea). To avoid false positives due to vaccination, only cats that had not received an FPV vaccine within the previous four weeks were included in the study (Patterson et al. 2007).

All enrolled cats underwent a physical examination, and demographic and environmental data, including age, sex, breed, vaccination status, ownership status, and lifestyle (indoor vs. outdoor access), were systematically recorded. Cats diagnosed with FPL were hospitalized for a period of five days for clinical monitoring and treatment. In order to determine the case-fatality rate, the entire study population was followed for 14 days. During this follow-up period, the survival status of discharged cats was confirmed *via* telephone interviews with the owners or scheduled recheck examinations.

At the time of admission, blood samples were collected from the cephalic vein of all enrolled cats. For blood gas analysis, plastic syringes containing sodium heparin were used, while tubes containing K_3_EDTA were used for CBC testing. Blood gas and CBC analyses were performed within 5–10 min of sample collection. For biochemical and biomarker analyses, blood samples were placed into plain tubes without anticoagulant, allowed to clot at room temperature for 15 min, and then centrifuged at 20 × g for 10 min. The obtained serum was divided into two aliquots: one was used immediately for biochemical analysis, and the other was stored at −80 °C for subsequent biomarker evaluation.

### Hematological and biochemical analyses

Venous blood pH, partial pressure of carbon dioxide (pCO_2_), partial pressure of oxygen (pO_2_), oxygen saturation (SO_2_), base deficit (BE), and bicarbonate (HCO_3_) were measured using an automated blood gas analyzer (ABL 90 Flex, Radiometer, Brea, CA, United States).

Total white blood cell (WBC), lymphocyte (Lym), monocyte (Mon), granulocyte (Gra), red blood cell (RBC), mean corpuscular volume (MCV), mean corpuscular hemoglobin concentration (MCHC), red blood cell distribution width (RDW), hematocrit (HCT), hemoglobin (Hb), and platelet count (PLT) were measured using an automated cell counter (Mindray BC-5000Vet, China).

Potassium (K), sodium (Na), calcium (Ca), chlorine (Cl), glucose (Glu), lactate (Lac) concentrations were measured using an automated blood gas analyzer (ABL 90 Flex, Radiometer, Brea, CA, United States). Blood urea nitrogen (BUN), creatinine (Cre), total bilirubin (Tbil), direct bilirubin (Dbil), alanine transaminase (ALT), aspartate aminotransferase (AST), alkaline phosphatase (ALP), lactate dehydrogenase (LDH), creatinine phosphokinase (CPK), gamma-glutamyl transferase (GGT), amylase (Amy), cholesterol (Chol), triglyceride (Tri), phosphorus (P), magnesium (Mg), total protein (Tpro), albumin (Alb), and albumin to globulin ratio (A: G) values were measured using an autoanalyzer (Biotecnica BT 3000 Plus, Italy).

Serum concentrations of ET-1 (Bioassay Technology Laboratory, Shanghai, China, Lot: E0095Cat), ADMA (Bioassay Technology Laboratory, Shanghai, China, Lot: E0092Cat), VEGF-A (SunRed Biotechnology Co., Ltd, Shanghai, China, Lot: 201-28-1439), and Syn-1 (Bioassay Technology Laboratory, Shanghai, China, Lot: E0068Cat) were measured using commercially available feline-specific enzyme-linked immunosorbent assay (ELISA) test kits according to the manufacturer’s instructions. The intra-assay coefficient of variation (CV), inter-assay CV, and minimum detectable concentrations (MDC) for the biomarkers were < 8%, < 10%, and 1.18 ng/mL for ET-1; < 8%, < 10%, and 0.014 nmol/mL for ADMA; < 10%, < 12%, and 1.864 pg/mL for VEGF-A; and < 8%, < 10%, and 0.025 ng/mL for Syn-1, respectively.

### Statistical analysis

Statistical analysis was performed using SPSS version 25.0 (IBM Corp., Armonk, NY, USA). The normality of the data distribution was assessed using the Shapiro-Wilks test. As the data were not normally distributed, all continuous variables were expressed as median and interquartile range (IQR). Comparisons between groups were performed using the Mann–Whitney U test. Pearson correlation coefficient was used to assess the correlation between variables. To determine the diagnostic and prognostic performance [cut-off values, sensitivity, specificity and, area under the curve (AUC)] of selected variables between survivors and non-survivors, receiver operating characteristic (ROC) curve analysis was conducted. A *P*-value of <0.05 was considered statistically significant.

## Results

### Demographic characteristics, clinical findings, and outcomes

Of the 30 cats diagnosed with FPL, 30% (*n* = 9) had a history of prior vaccination, while 70% (*n* = 21) were unvaccinated. The age of the cats ranged from 6 weeks to 5 years. Seventeen cats were male (56.7%) and thirteen were female (43.3%). Breed distribution included 11 Scottish Fold, 7 British Shorthair, 5 Persian, 4 Domestic Shorthair, and 3 Siamese cats. All cats were owned; except for five individuals, the majority had access to the outdoors.

The most commonly observed clinical signs in FPL-affected cats were anorexia, vomiting, lethargy, and diarrhea. Hemorrhagic diarrhea was noted in only two cases. Hyperthermia was observed in 15 cats, while 5 cats exhibited hypothermia. A total of 63.3% (*n* = 19) of the cats responded to treatment and survived, whereas 36.7% (*n* = 11) died during the course of treatment. Regarding the timeline of mortality, one cat died on the first day, six on the third day, and four on the fifth day. The average time to death was calculated as 3.55 days.

The control group consisted of 10 healthy cats between 2 months and 5 years of age. This group included 6 males and 4 females. Breed distribution comprised 4 Domestic Shorthair (Tabby), 2 Persian, 2 British Shorthair, 1 Siamese, and 1 Scottish Fold. All control cats were owned and lived exclusively indoors. Five cats were fully vaccinated, three were unvaccinated, and vaccination records were not available for the remaining two cats. No abnormalities were detected during clinical examination, and all results from CBC, blood gas analysis, and biochemical testing were within reference ranges.

### Hematological and biochemical findings

Blood gas and hemogram results of healthy and cats with FPL are shown in Table 1. While pH, Ca, BE and HCO_3_ levels were significantly lower in cats with FPL compared to healthy cats, Lac concentration was higher (*p* < 0.05). In CBC, WBC, Lym, Mon, Gra, MCHC and PLT levels were significantly lower in cats with FPL than in healthy cats (*p* < 0.05).

**Table 1.** Venous blood gas and complete blood count results of healthy and cats with panleukopenia.

Variable	Healthy (*n* = 10)	Panleukopenia (*n* = 30)	*p* Value	Ref. range
pH	7.40 (7.38-7.42)	7.34 (7.31-7.41)	0.04	7.244–7.444
pCO_2_ (mmHg)	29.90 (21.90-36.20)	28.75 (23.40-34.65)	0.79	27.3–49.1
pO_2_ (mmHg)	33.75 (30.45-38.05)	36.25 (33.20-40.00)	0.14	33.9–56.3
SO_2_ (%)	52.85 (46.75-65.95)	52.90 (44.20-60.45)	0.69	44.50-71.10
K (mmol/L)	3.90 (3.25-4.30)	3.60 (3.40-3.90)	0.31	3.11–4.64
Na (mmol/L)	154.00 (149.25-157.50)	153.00 (151.00-157.50)	0.96	150.5–157.2
Ca (mmol/L)	1.24 (1.23-1.42)	1.28 (1.20-1.40)	0.01	1.21–1.45
Cl (mmol/L)	117.50 (112.75-123.25)	120.50 (117.50-123.00)	0.18	113–123
Glukoz (mg/dL)	104.00 (85.50-126.50)	126.00 (82.00-153.00)	0.36	70-150
Lac (mmol/L)	1.60 (1.20 − 1.70)	1.95 (1.60-3.15)	0.01	0.61–5.86
BE (mmol/L)	−3.45 (-3.93 - −1.98)	−9.65 (-11.85 - −7.35)	<0.001	−10.4 − 0.5
HCO_3_ (mmol/L)	19.30 (18.20 − 21.75)	16.90 (15.30-18.15)	<0.001	15.8- 23.4
WBC (cells/mL)	12.34 (9.33-14.35)	3.21 (2.40-3.88)	<0.001	5.0-19.00
Lym (cells/mL)	3.80 (2.36-5.82)	2.01 (0.95-2.82)	0.01	0.2-5.7
Mon (cells/mL)	1.12 (0.45-2.34)	0.12 (0.08-0.37)	<0.001	0.1-1.1
Gra (cells/mL)	7.06 (4.23-9.57)	0.56 (0.29-1.25)	<0.001	2.0-15.2
RBC (×10^3^ cells/mL)	10.97 (9.04-11.81)	10.26 (7.39-11.58)	0.28	4.0-9.0
MCV (fL)	46.70 (43.20-54.43)	49.80 (47.05-51.95)	0.39	35.5-55.0
MCHC (g/dL)	27.45 (25.75-28.10)	24.65 (22.65-28.10)	0.02	28.0-40.0
Hct (%)	48.05 (43.38-57.43)	47.85 (36.25-57.55)	0.48	24.0–45.0
Hb (g/dL)	13.45 (11.28-15.48)	11.20 (10.30-13.45)	0.07	9.5–15.0
PLT (cells/mL)	152.00 (128.25-197.00)	57.00 (39.50-89.00)	<0.001	120-500

Data shown as median [IQR].

Partial carbon dioxide pressure (pCO_2_), partial oxygen pressure (pO_2_), oxygen saturation (SO_2_), potassium (K), sodium (Na), calcium (Ca), chlorine (Cl), glucose (Glu), lactate (Lac), base deficit (BE), bicarbonate (HCO_3_), total leukocytes (WBC), lymphocytes (Lym), monocytes (Mon), granulocytes (Gra), erythrocytes (RBC), mean corpuscular volume (MCV), mean corpuscular hemoglobin concentration (MCHC), hematocrit (HCT), hemoglobin (Hb), platelet count (PLT).

Results of biochemical analysis of healthy cats and cats with FPL are shown in Table 2. TBil and DBil levels were significantly higher in cats with FPL than in healthy cats (*p* < 0.05). There was no statistically significant difference in other biochemical analysis parameters (*p* > 0.05).

**Table 2.** Biochemical parameters of healthy and cats with panleukopenia.

Variable	Healthy (*n* = 10)	Panleukopenia (*n* = 30)	*P* value	Ref. range
BUN (mg/dL)	19.50 (16.63-22.08)	17.90 (14.15-23.85)	0.72	4.7-34.0
Cr (mg/dL)	1.25 (0.88-1.43)	0.95 (0.76-1.27)	0.25	0.80-1.80
ALT (U/L)	47.50 (27.75-59.75)	55.00 (33.28-103.50)	0.39	10.0-80.0
AST (U/L)	29.00 (16.16-51.50)	38.50 (29.50-63.00)	0.10	10.0-80.0
ALP (U/L)	38.50 (25.75-52.25)	34.50 (20.00-88.50)	0.79	10.0-80.0
GGT (U/L)	3.00 (1.75-5.00)	2.00 (1.00-3.33)	0.18	1.0-10.0
Chol (mg/dL)	143.00 (99.75-219.75)	153.50 (127.35-176.59)	0.67	90.0-205.0
Tri (mg/dL)	39.50 (29.75-53.00)	44.00 (29.37-78.50)	0.48	10.0-114.0
Tbil (mg/dL)	0.45 (0.28-1.01)	1.20 (0.67-2.27)	0.001	0.1-0.6
Dbil (mg/dL)	0.20 (0.18-0.40)	0.52 (0.30-1.00)	0.01	0.0-0.3
Amy (U/L)	1266.00 (1083.00-1591.25)	1050.00 (685.18-1807.50)	0.33	500.0-1800
LDH (U/L)	161.50 (90.00-463.75)	296.00 (158.50-606.43)	0.28	75.0-490.0
CPK (U/L)	464.00 (206.00-566.75)	284.50 (131.00-470.27)	0.67	50.0-450.0
ALB (g/dL)	3.25 (2.78-4.00)	3.42 (3.08-3.87)	0.56	2.1-3.9
Tpro (g/dL)	6.85 (6.35-7.61)	6.65 (6.10-7.53)	0.56	5.4-7.8
A:G	0.84 (0.60-1.42)	1.06 (0.86-1.30)	0.30	–
Mg (mg/dL)	2.30 (1.88-2.53)	2.10 (1.85-2.30)	0.22	1.5-3.5
P (mg/dL)	5.70 (3.85-6.43)	4.80 (3.70-6.03)	0.22	1.8-6.4

Data shown as median [IQR].

Blood urea nitrogen (BUN), creatinine (Cr), alanine transaminase (ALT), aspartate aminotransferase (AST), alkaline phostatase (ALP), gamma glutamyl transferase (GGT), cholesterol (Chol), triglycerides (Tri), total bilirubin (Tbil), direct bilirubin (Dbil), amylase (Amy), lactate dehydrogenase (LDH), creatine phosphokinase (CPK), albumin (Alb), total protein (Tpro), albumin globulin ratio (A: G), magnesium (Mg), phosphorus (P).

### eGCx biomarker findings

Biomarker concentrations of healthy cats and cats with FPL are shown in Table 3. ET-1, ADMA, VEGF-A, and Syn-1 concentrations in cats with FPL were significantly higher than in the control group (*p* < 0.01).

**Table 3.** Endothelial glycocalyx biomarker concentration findings in healthy and cats with panleukopenia. Data shown as median [IQR].

Variable	Healthy (*n* = 10)	Panlekopenia (*n* = 30)	*P* value
ET-1 (ng/L)	36.57 (25.76-46.82)	81.52 (68.42-93.29)	<0.001
ADMA (nmol/mL)	1.38 (1.22-1.98)	2.00 (1.76-2.53)	0.002
VEGF-A (pg/mL)	35.35 (25.89-54.79)	81.52 (74.28-92.38)	<0.001
Syn-1 (ng/mL)	2.50 (2.13-2.81)	3.00 (2.66-3.21)	0.008

Endothelin-1 (ET-1), asymmetric dimethylarginine (ADMA), vascular endothelial growth factor-A (VEGF-A), syndecan-1 (Syn-1).

### Associations between variables

Correlations between eGCx biomarker concentrations and some hematological parameters in healthy and cats with FPL are shown in Table 4. Blood Lac concentration showed a weak negative correlation with PLT (*p* < 0.01). WBC concentration showed a moderate positive correlation with PLT, while ET-1 and VEGF-A concentrations showed a moderate negative correlation (*p* < 0.01). RBC level showed weak positive correlation with PLT and weak negative correlation with Syn-1 (*p* < 0.01). PLT level showed moderate negative correlation with ET-1 (*p* < 0.01) and weak negative correlation with VEGF-A and Syn-1 (*p* < 0.05). Serum ET-1 concentration was moderately positively correlated with VEGF-A and ADMA and strongly and positively correlated with serum Syn-1 concentration (*p* < 0.01). There was a weak positive correlation between VEGF-A and blood serum Syn-1 and ADMA concentrations (*p* < 0.05). Blood serum Syn-1 concentration was moderately and strongly correlated with serum ADMA concentration (*p* < 0.01).

**Table 4.** Pearson correlations between the concentrations of biomarkers of the endothelial glycocalyx and some hematological parameters in healthy and cats with panleukopenia.

Variable	Lac	WBC	RBC	PLT	ET-1	VEGF-A	Syn-1	ADMA
Lac	1	−0.23	0.23	−0.40**	0.27	0.20	0.01	0.25
WBC		1	0.09	0.52**	−0.56**	−0.63**	−0.25	−0.30
RBC			1	0.42**	−0.29	−0.03	−0.41**	−0.14
PLT				1	−0.54**	−0.31*	−0.32*	−0.24
ET-1					1	0.52**	0.76**	0.44**
VEGF-A						1	0.32*	0.33*
Syn-1							1	0.57**
ADMA								1

Lactate (Lac), total leukocytes (WBC), erythrocytes (RBC), platelet count (PLT), endothelin-1 (ET-1), vascular endothelial growth factor-A (VEGF-A), syndecan-1 (Syn-1), asymmetric dimethylarginine (ADMA).

** Correlation is significant at the 0.01 level.

* Correlation is significant at the 0.05 level.

### Prognostic performance of biomarkers

**Figure 1.**
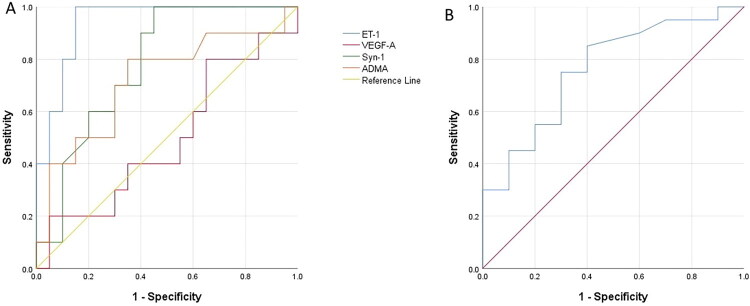
Receiver operating characteristic (ROC) curve analysis to discriminate between surviving and non-surviving cats with panleukopenia based on (A) serum endothelial glycocalyx concentrations and (B) blood monocyte counts.

The results of ROC analysis performed to determine the relationship between eGCx biomarkers and some blood parameters and mortality in non-surviving cats with FPL are presented in Table 5 and Figure 1. As a result of the ROC analysis, the area AUC at the ET-1 cut-off value of 86.14 ng/L was 0.940 (95% confidence interval (CI) 0.859-1.000; *p* = 0.000) with 100% sensitivity and 85% specificity; AUC 0.780 (95% CI: 0.617-0.943; *p* = 0.014), with 70% sensitivity and specificity; AUC at Mon cut-off 0.10 (cells/mL) was 0.763 (95% CI: 0.581-0.944; *p* = 0.021), with 75% sensitivity and 70% specificity, were found to be good prognostic indicators for predicting mortality in cats with panleukopenia.

**Table 5.** Area under the curve (AUC), standard error, confidence interval (95%), optimal cut-off values, and corresponding sensitivity and specificity values for predicting mortality in non-surviving cats with panleukopenia.

Variable	AUC	Standard Error	*P-*Value	Asymptotic 95% Confidence Interval		Sensitivity	Specificity	Cut-Off Value
				Lower Band	Upper Bound			
ET-1	0.940	0.041	<0.001	0.859	1.000	100	85	86.14
ADMA	0.718	0.106	0.056	0.510	0.925	80	65	2.00
VEGF-A	0.495	0.116	0.965	0.268	0.722	50	45	80.43
Syn-1	0.780	0.083	0.014	0.617	0.943	70	70	3.04
Mon	0.763	0.093	0.021	0.581	0.944	75	70	0.10

Endothelin-1 (ET-1), asymmetric dimethylarginine (ADMA), vascular endothelial growth factor-A (VEGF-A), syndecan-1 (Syn-1), monocytes (Mon).

## Discussion

In this study, serum concentrations of ET-1, ADMA, VEGF-A, and Syn-1, which are markers of eGCx damage, were evaluated in cats with panleukopenia. Our results showed that all eGCx damage biomarker concentrations were high and positively correlated with each other in cats with panleukopenia. In addition, high serum concentrations of ET-1 and Syn-1 were significant predictors of mortality in cats with panleukopenia and may therefore be useful as prognostic markers. In conclusion, our results suggest that endothelial and microvascular dysfunction occurs in cats with panleukopenia and that glycocalyx damage may play a role in the pathogenesis of the disease.

Due to the virus’s marked tropism for rapidly dividing cells, including intestinal crypt epithelia, lymphoid tissues, and bone marrow, acid-base and fluid-electrolyte disturbances associated with vomiting and diarrhea are inevitable in infected cats. However, there are no studies evaluating blood gases in cats with panleukopenia. This study provides the first detailed evaluation of blood gas parameters and acid–base disturbances in cats with FPL. pH, Ca, BE, and HCO_3_ levels were significantly lower in affected cats than in healthy cats, while Lac concentration was higher. These findings indicate that significant changes in blood gases and acid–base balance (hyperlactatemia and metabolic acidosis) are most likely due to HCO_3_loss in diarrhea and D-lactate production by intestinal bacteria. Similar alterations in blood gases have also been reported in parvovirus-infected dogs (Nappert et al. 2002; Goddard and Leisewitz 2010; Gulersoy et al. 2022).

Leukopenia, characterized by neutropenia, lymphopenia, and monocytopenia, is the most common finding in cats with panleukopenia (Porporato et al. 2018; Gulersoy et al. 2023a, 2023b). These hematologic changes likely result from the cytopathic effects of FPV on the bone marrow and the redistribution or loss of leukocytes during early infection and sepsis (Greene 2012; Stuetzer and Hartmann 2014; Belok et al. 2021; Pandey 2022). Monocytopenia, an indicator of poor prognosis, was commonly observed in this study and, with an AUC of 0.763 at a cut-off of 0.10 cells/mL, predicted mortality with 75% sensitivity and 70% specificity. Previous studies also report that neutropenia, lymphopenia, monocytopenia, and leukopenia at presentation are prognostic indicators of poor outcome in FPL (Kruse et al. 2010; Gulersoy et al. 2023a). Evaluation of the leukogram at admission can therefore aid diagnosis, and Mon levels can serve as a useful prognostic marker. The other major hematologic abnormality observed in our study was thrombocytopenia. Thrombocytopenia has been reported to be a variable feature of FPL and may coexist with other coagulation abnormalities in cats with megakaryocyte destruction or DIC (Kruse et al. 2010). However, it has also been reported that it may be associated with leukopenia in the early stages of infection that is directly related to damage to the bone marrow (Greene 2012). It is also important to consider that feline PLT measured *via* automated hematology analyzers can be underestimated due to frequent platelet clumping, potentially resulting in spurious thrombocytopenia. Although it is thought that bone marrow suppression or sepsis-related inflammatory disease anemia may develop in cats with FPL, anemia did not occur unexpectedly in our study. It is suggested that the cats with panleukopenia included in the study did not develop anemia because of the sudden onset of the disease, the absence of hemorrhagic diarrhea, and the relatively long lifespan of erythrocytes (Greene 2012).

Changes in serum biochemistry in FPV infection are usually non-specific. It has been reported that mild to moderate increases in ALT and AST activities or bilirubin concentration may reflect hepatic involvement, but icterus is a rare finding (Greene 2012). The elevated bilirubin levels observed in cats with FPV infection likely reflect indirect hepatic involvement, resulting from systemic effects such as sepsis, reduced hepatic perfusion, or DIC, rather than direct viral-induced hepatocellular damage, as FPV does not primarily target hepatic tissue (Greene 2012; Stuetzer and Hartmann 2014). In the present study, the high levels of Tbil and Dbil in cats with panleukopenia suggest that FPV probably causes liver dysfunction (Greene 2012; Gülersoy et al. 2023b).

In inflammatory conditions such as FPL, endothelial dysfunction may be accompanied by disruption of the eGCx, which in turn exacerbates inflammation and contributes to disease progression (Qu et al. 2021). Indeed, glycocalyx degradation is considered an early step in the inflammatory cascade (Lupu et al. 2020). Recent studies have highlighted the diagnostic and prognostic relevance of eGCx-associated biomarkers in veterinary medicine (Naseri et al. 2020; Ider et al. 2024). To the best of our knowledge, this is the first study to assess circulating concentrations of ET-1, ADMA, VEGF-A, and Syn-1 as indicators of endothelial injury in cats with FPL.

ET-1, a vasoconstrictor peptide produced by endothelial cells (Kedzierski & Yanagisawa 2001; Masaki & Sawamura 2006; Xu et al. 2023), has been shown to contribute to the development of inflammatory processes in the vascular wall (Kowalczyk et al. 2015) and to induce extensive intravascular coagulation in sepsis (Kapiotis et al. 1997). High concentrations of ET-1 have been associated with endothelial dysfunction in cats with hemotropic mycoplasmosis (Ider et al. 2024) and in humans with sepsis (Kowalczyk et al. 2015) as a result of reduced NO bioavailability. In addition, ET-1 induces glycocalyx disruption by inducing the release of heparanase, which degrades the heparan sulfate side chains that form the basic structure of eGCx (Cao et al. 2019; Ider et al. 2024). In the present study, the significantly elevated ET-1 concentrations observed in cats with panleukopenia compared to healthy controls may reflect glycocalyx damage and endothelial dysfunction, potentially mediated by ET-1 induced heparanase activation (Cao et al. 2019; Ider et al. 2024) and reduced NO bioavailability (Xu et al. 2023; Ider et al. 2024). In addition, the negative correlations of this biomarker with WBC and PLT levels suggest that ET-1 plays a proinflammatory (Kowalczyk et al. 2015) and procoagulant (Kapiotis et al. 1997) role in the pathogenesis of panleukopenia.

ADMA, an endogenous inhibitor of NOs, contributes to both acute and chronic endothelial dysfunction by reducing NO bioavailability (Davis et al. 2011). In human sepsis, elevated plasma ADMA concentrations have been associated with organ dysfunction (O’Dwyer et al. 2006; Nakamura et al. 2009; Iapichino et al. 2010). This effect is thought to result from decreased constitutive endothelial NO production, which is essential for vascular homeostasis. Moreover, ADMA has been reported to induce heparanase, a key ‘cardenase’ implicated in glycocalyx degradation, thereby further contributing to endothelial injury (Garsen et al. 2016; Lukasz et al. 2017; Barber et al. 2021; Ider et al. 2024). In the present study, the higher serum ADMA concentrations in cats with panleukopenia compared to the control group suggest impaired endothelial and microvascular function due to decreased endothelial NO production in cats with panleukopenia and thus the development of glycocalyx damage (O’Dwyer et al. 2006; Nakamura et al. 2009; Iapichino et al. 2010; Davis et al. 2011). In addition, the moderate positive correlation between serum ET-1 and ADMA may indicate that heparanase activation in panleukopenic cats (Garsen et al. 2016; Lukasz et al. 2017; Barber et al. 2021; Ider et al. 2024) appears to contribute to the development of eGCx damage.

VEGF-A plays a major role in increasing vascular permeability, an important pathophysiologic mechanism of sepsis, it has been suggested that it may better reflect the mechanism of sepsis progression than other biomarkers (Tang et al. 2022). In humans with sepsis, VEGF-A concentrations have been found to increase in association with endothelial dysfunction (Van der Flier et al. 2005; Karlsson et al. 2008; Jurisic and Detmar 2009; Pons et al. 2020). It has been shown that VEGF-A concentrations are higher in dogs with sepsis compared to healthy dogs and that endothelial activation occurs in dogs with sepsis similar to humans (König et al. 2019; Gaudette et al. 2023). However, in inflammatory diseases, VEGF-A has been reported to exert a protective effect on endothelial cells against decreased NO bioavailability by stimulating NO release and upregulating NOs expression (Karlsson et al. 2008; Hanson et al. 2015). In the present study, the high concentrations of VEGF-A and the negative correlation of this biomarker with WBC and PLT in cats with panleukopenia compared to healthy cats suggest that endothelial dysfunction occurs similarly to dogs and humans with sepsis. However, we suggest that VEGF-A is elevated in cats with panleukopenia due to the protective effects of VEGF-A (Karlsson et al. 2008; Hanson et al. 2015) against decreased NO bioavailability (increased ET and ADMA concentrations).

Syn-1, an important structural component of the eGCx, plays a key role in endothelial function (Ikeda et al. 2018). There is increasing evidence that Syn-1 is degraded by various mediators such as cytokines or reactive oxygen species during inflammatory conditions and that its high concentrations in the circulation are a sensitive indicator of endothelial dysfunction and glycocalyx damage (van Golen et al. 2012; Anand et al. 2016; Ider et al. 2024). In the present study, higher concentrations of Syn-1 in cats with panleukopenia compared to healthy cats were associated with the development of endothelial dysfunction and glycocalyx damage.

Syn-1 concentrations have been reported to negatively correlate with the glycocalyx stabilizer sphingosine-1-phosphate (S-1-P) and PLT in sepsis patients, suggesting that elevated Syn-1 reflects consumptive coagulopathy rather than intravascular coagulation activation (Ikeda et al. 2018; Hatanaka et al. 2021; Piotti et al. 2021). Similar associations have been observed in our previous study in cats with hemotropic mycoplasmosis (Ider et al. 2024) and in humans with malaria (Barber et al. 2021), where Syn-1 concentrations were inversely related to RBC and PLT, likely due to S-1-P depletion resulting from anemia and/or thrombocytopenia. In the present study, the negative correlations between Syn-1, RBC, and PLT may reflect the same mechanism of glycocalyx disruption. However, the observed inverse relationship between ET-1, Syn-1, and PLT in cats with panleukopenia may also be attributable to the pancytopenic effects of FPV infection itself (Pandey, 2022), rather than consumptive coagulopathy (Hatanaka et al. 2021).

FPL is a highly fatal disease, with reported mortality rates ranging from 33% to 49% (Kruse et al. 2010; Petini et al. 2020; Safwat et al. 2024) and an average survival time of 2 to 3.5 days among non-surviving cats, despite supportive treatment (Kruse et al. 2010; Porporato et al. 2018; Petini et al. 2020). In the present study, the observed case-fatality rate was 36.7%, and the mean survival time among non-surviving cats was 3.55 days. This finding is largely consistent with previously reported survival durations of 3 days (Porporato et al. 2018) and 3.5 days (Petini et al. 2020). The high case-fatality rates and short survival times clearly highlight the devastating nature of FPL, which often leads to rapid death due to complications such as sepsis, hypovolemic shock, and DIC (Barrs 2019). Nonetheless, prognosis in cats with panleukopenia has been associated with various clinical (hypothermia, lethargy, and low body weight) and laboratory parameters (low leukocyte, Mon, Alb, K, and total thyroxine levels) (Kruse et al. 2010; Porporato et al. 2018; Petini et al. 2020). Although eGCx damage has been reported to be associated with mortality in several diseases (Pan et al. 2012), these markers have not been evaluated in cats with panleukopenia. Therefore, in accordance with the objectives of our study, the usefulness of eGCx markers in predicting prognosis was evaluated. In the present study, ET-1 cut-off > 86.14 ng/L (100% sensitivity and 85% specificity) and Syn-1 cut-off > 3.04 ng/mL (70% sensitivity and 70% specificity) were found to be important indicators for predicting mortality in cats with panleukopenia. Therefore, evaluation of ET-1 and Syn-1 together with previously described prognostic clinical and laboratory parameters in cats with panleukopenia may be useful in clinical practice.

The present study has several limitations. First, the relatively small number of cats included in each study group limits the generalizability of our findings. Second, blood samples were collected only at the time of admission; therefore, longitudinal data were not available to assess the dynamic progression of eGCx damage and its long-term impact on organ dysfunction and mortality. Third, FPV screening in the control group relied on rapid antigen tests, which have limited sensitivity in asymptomatic cats; consequently, subclinical infections may not have been completely excluded. The use of a more sensitive method such as qPCR could have improved diagnostic accuracy and ensured greater integrity of the control group. Fourth, although eGCx injury was evaluated using cat-specific ELISA assays, no histopathologic confirmation was performed, and in-house validation of the assays (e.g. spike-and-recovery or reproducibility testing) was not conducted, which may affect the robustness of the biomarker measurements. Finally, while some biomarkers such as ET-1 and Syn-1 demonstrated prognostic potential, these assays are currently available only in research settings, are costly, and are not yet applicable as point-of-care diagnostic tools in clinical practice.

## Conclusion

In our study, the concentrations of all eGCx biomarkers (Syn-1, ET-1, ADMA, VEGF-A) were high and positively correlated with each other in cats with panleukopenia. In addition, Syn-1 and ET-1 were found to be potential prognostic factors with high sensitivity and specificity. These findings suggest that endothelial and microvascular functions are impaired and eGCx damage develops in cats with panleukopenia due to decreased endothelial NO bioavailability. In addition, the results of this study suggest that endothelial dysfunction and eGCx damage play a role in the pathogenesis of panleukopenia.

## Data Availability

All data generated or analyzed during the current study are included in this article. The data supporting the findings of this study are available from the corresponding author, upon reasonable request.
